# Preliminary exploration of radiotherapy timing following induction chemoimmunotherapy in unresectable locally advanced esophageal squamous cell carcinoma: a single-center retrospective study

**DOI:** 10.3389/fonc.2025.1703563

**Published:** 2025-12-15

**Authors:** Aiju Zeng, Xianquan Zhang, Tian Xu, Yu Zhang, Daiyuan Ma

**Affiliations:** 1 Department of Oncology, Affiliated Hospital of North Sichuan Medical College, Nanchong, China; 2 Department of Thoracic Surgery, The Affiliated Nanchong Central Hospital of North Sichuan Medical College, Nanchong, China

**Keywords:** esophageal squamous cell carcinoma, cancer immunotherapy, chemotherapy, radiotherapy, timing of intervention

## Abstract

**Objective:**

To evaluate the efficacy and safety of the sequential strategy involving induction chemoimmunotherapy followed by radiotherapy for locally advanced unresectable esophageal squamous cell carcinoma (ESCC), and to explore the optimal timing for radiotherapy intervention and the potential clinical benefits of subsequent consolidative immunotherapy.

**Method:**

This study retrospectively collected clinical data from treatment-naïve patients with unresectable T1-4N0-3M0 stage ESCC who underwent induction chemoimmunotherapy followed by radiotherapy at the Affiliated Hospital of North Sichuan Medical College between December 2021 and May 2024. Patients were stratified into two cohorts: the early radiotherapy cohort (early-RT, n=34), who initiated radiotherapy after completion of 1–2 cycles of induction chemoimmunotherapy, and the late radiotherapy cohort (late-RT, n=44), who initiated radiotherapy after completing 3–6 cycles of induction chemoimmunotherapy. The primary endpoints were progression-free survival (PFS) and overall survival (OS), with safety serving as the secondary endpoint.

**Result:**

With a cutoff date of May 31, 2025, the median follow-up duration was 25.9 months. In the overall population, the median PFS was 16.0 months (95% confidence interval [CI], 11.7-20.3) and the median OS was 25.0 months (95%CI, 21.49-28.51). PFS was significantly longer in the early-RT cohort than in the late-RT cohort(20.4 vs. 13.7 months; HR = 0.53; *p* = 0.032). No statistically significant difference in OS was observed between the two cohorts (26.6 vs. 24.8 months; *p* = 0.728). Subsequent consolidative immunotherapy significantly improved PFS (median not reached [NR] vs. 12.8 months; HR = 0.46; *p* = 0.011) and OS (NR vs. 24.1 months; HR = 0.43; *p* = 0.017). In terms of safety, most treatment-related adverse events (TRAEs) were grade 1 or 2 in severity and were manageable. The incidence of toxicities did not differ significantly between the early-RT and late-RT cohorts (*p* > 0.05).

**Conclusion:**

In patients with unresectable locally advanced ESCC, the combination of early radiotherapy intervention and subsequent consolidative immunotherapy may represent a potential preferred therapeutic strategy. The findings from this study provide a rationale for the design of phase III clinical trials.

## 1 Introduction

Esophageal cancer (EC) is a highly prevalent malignant tumor originating from the epithelial lining of the digestive system. Global cancer statistics from 2022 indicate that esophageal cancer is the 11th most frequently diagnosed cancer and the 7th leading cause of cancer death worldwide (1). In China, esophageal squamous cell carcinoma (ESCC) is the predominant histological type. Due to its frequently asymptomatic nature in the early stages, most patients are diagnosed with locally advanced disease that is unsuitable for curative surgery at initial presentation (2, 3). The RTOG 85–01 trial established definitive concurrent chemoradiotherapy (dCRT) as the standard treatment modality for patients with unresectable locally advanced esophageal squamous cell carcinoma (ESCC) (4). However, treatment outcomes are unsatisfactory. Despite receiving standard dCRT, patients still face a persistently high risk of disease progression, recurrence, or metastasis, with a 5-year overall survival rate remaining below 30% (5). There is an urgent unmet need for the development of more effective therapeutic regimens to improve clinical outcomes for these patients.

The recent clinical integration of immune checkpoint inhibitors (ICIs) has accelerated research into their role within the multimodal management of esophageal cancer. In the context of unresectable locally advanced ESCC, key modalities for immunotherapy integration encompass: the induction phase (chemotherapy combined with immunotherapy) (6–9), the concurrent phase (chemoradiotherapy combined with immunotherapy) (10–15), and the consolidation phase (maintenance immunotherapy following radiotherapy (11, 13, 14, 16). Current research has largely focused on concurrent and consolidative immunotherapy, while clinical evidence supporting the sequential strategy of induction chemoimmunotherapy followed by radiotherapy remains relatively scarce. Moreover, the optimal timing for radiotherapy intervention within this sequence and the clinical necessity of subsequent consolidative immunotherapy have not been systematically evaluated, representing significant knowledge gaps in this field.

Hence, the present study was designed to investigate the optimal timing of radiotherapy after induction chemoimmunotherapy and the potential clinical benefit of subsequent consolidative immunotherapy, with the goal of optimizing the multimodal management strategy for patients with locally advanced ESCC.

## 2 Materials and methods

### 2.1 Study population

**Figure 1:**
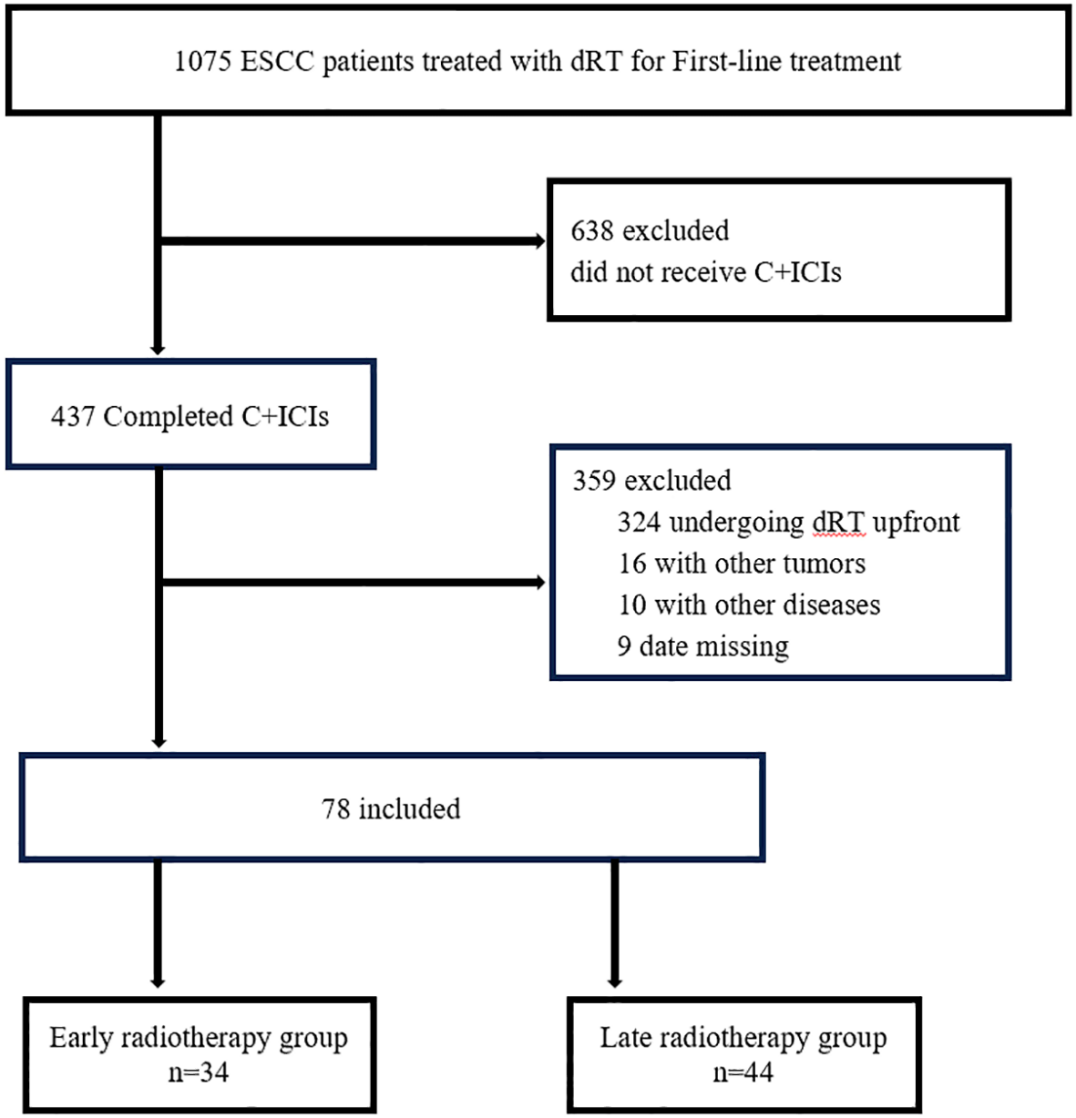
Patient screening flowchart.

Patients with unresectable locally advanced ESCC who underwent induction chemoimmunotherapy followed by radiotherapy at the Affiliated Hospital of North Sichuan Medical College between December 2021 and May 2024 were retrospectively enrolled. The cutoff date for follow-up was May 31, 2025. Inclusion criteria were: (1) Histologically confirmed esophageal squamous cell carcinoma (ESCC); (2) Clinical stage T1-4N0-3M0 (AJCC 8th edition), deemed unresectable following assessment by a multidisciplinary team (MDT); (3)Received first-line chemoimmunotherapy for 4 to 6 cycles; (4) Underwent local radiotherapy to the primary tumor site (total dose ≥50 Gy) during the course of systemic therapy (within the 4–6 cycles). Exclusion criteria were as follows: (1) History of or concurrent other active malignancies; (2) Severe dysfunction or failure of major organs, including significant cardiovascular or cerebrovascular disease, hepatic insufficiency, or renal impairment; (3) Active autoimmune disorders or infectious diseases. (**Figure 1**).

This study was approved by the Ethics Committee of the Affiliated Hospital of North Sichuan Medical College (Approval No.: 2025ER402-1) and was conducted in accordance with the principles of the Declaration of Helsinki (2013).

### 2.2 Method

All patients received first-line induction chemoimmunotherapy followed by definitive radiotherapy, with or without subsequent immune consolidation. Patients receiving radiotherapy after 1–2 cycles of induction chemoimmunotherapy were classified as the early radiotherapy group, while those receiving radiotherapy after 3–6 cycles were classified as the late radiotherapy group. The induction chemotherapy regimen consisted of a platinum-based doublet combined with a taxane (paclitaxel or nab-paclitaxel[175 mg/m², intravenous infusion]), with anti-PD-1/PD-L1 agents (Sintilimab 200 mg, intravenous infusion; Toripalimab 200 mg, intravenous infusion; Camrelizumab 200 mg, intravenous infusion; Tislelizumab 200 mg, intravenous infusion). Radiotherapy was delivered using intensity-modulated radiation therapy (IMRT), with concurrent chemotherapy consisting S-1 monotherapy(40 mg orally twice daily).

### 2.3 Results and statistical analysis

The primary endpoints were progression-free survival (PFS) and overall survival (OS). PFS was defined as the time from the initiation of induction chemoimmunotherapy to the first occurrence of disease progression, death from any cause, or the last follow-up. OS was defined as the time from the start of induction chemoimmunotherapy to death from any cause or the last follow-up. The secondary endpoint was safety. Treatment-related adverse events (TRAEs) were graded using the Common Terminology Criteria for Adverse Events (CTCAE) version 5.0. Statistical analyses were performed using SPSS software (version 26.0). Categorical data are presented as number (n) and percentage (%). Baseline characteristics were compared using the Chi-square test or Fisher’s exact test, as appropriate. Median follow-up time was estimated by the Reverse Kaplan-Meier method. Survival curves were generated using the Kaplan-Meier method and compared using the log-rank test. Univariate and multivariate Cox proportional hazards regression models were used for prognostic analysis. Survival curves and forest plots for prognostic factors were constructed using GraphPad Prism software (version 10.0). A two-sided p-value < 0.05 was considered statistically significant.

## 3 Results

### 3.1 Baseline characteristics

Between December 2021 and May 2024, 78 treatment-naïve patients with unresectable locally advanced ESCC were enrolled. Thirty-four patients (43.59%) were allocated to the early radiotherapy group, and 44 patients (56.41%) were allocated to the late radiotherapy group. The cohort included 56 males (71.79%), and 67 patients (85.90%) had stage III disease at initial diagnosis. All patients received a median of 3 cycles (range, 2-3) of induction chemoimmunotherapy, followed by either concurrent chemoradiotherapy (CCRT) or definitive radiotherapy. Immune consolidation therapy was administered to 25 patients (32.05%), including 12 patients in the early group and 13 in the late group. Baseline characteristics of the two groups are summarized in **Table 1**. No significant differences were observed between the two groups in terms of gender, age, smoking status, alcohol consumption, ECOG performance status, tumor location, clinical stage, radiotherapy dose, or immune consolidation (*p* > 0.05). (**Table 1**).

**Table 1:** Baseline characteristics of patients in the early and late radiotherapy group.

Feature	Early radiotherapy group(n=34) n(%)	Late radiotherapy group(n=44) n(%)	*X* ^2^	*p* value
Gender				
Male	23(67.65)	33(75.00)	0.512	0.474
Female	11(32.45)	11(25.00)		
Age				
<65	13(38.24)	17(38.66)	0.001	0.971
≥65	21(61.76)	27(61.36)		
Smoking				
Before or now	17(50.00)	21(47.73)	0.040	0.842
Never	17(50.00)	23(52.27)		
Drinking				
Before or now	12(35.29)	19(43.18)	0.498	0.480
Never	22(64.71)	25(56.82)		
ECOG				
0-1	33(97.06)	42(95.45)	0.000	1.000
2	1(2.94)	2(4.55)		
Tumor location				
Upper	6(17.65)	8(18.18)	2.239	0.524
Middle	17(50.00)	16(36.36)		
Lower	6(17.65)	8(18.18)		
Multi-site	5(14.70)	12(27.28)		
Stage				
IIIA	2(5.88)	5(11.36)	1.164	0.570
IIIB	26(76.47)	34(77.27)		
IVA	6(17.65)	5(11.37)		
Radiotherapy dose				
<60Gy	9(26.47)	16(36.36)	0.862	0.353
≥60Gy	25(73.53)	28(63.64)		
Immune consolidation				
Yes	12(35.29)	13(29.55)	0.291	0.590
No	22(64.71)	31(70.45)		

### 3.2 Survival outcomes

**Figure 2:**
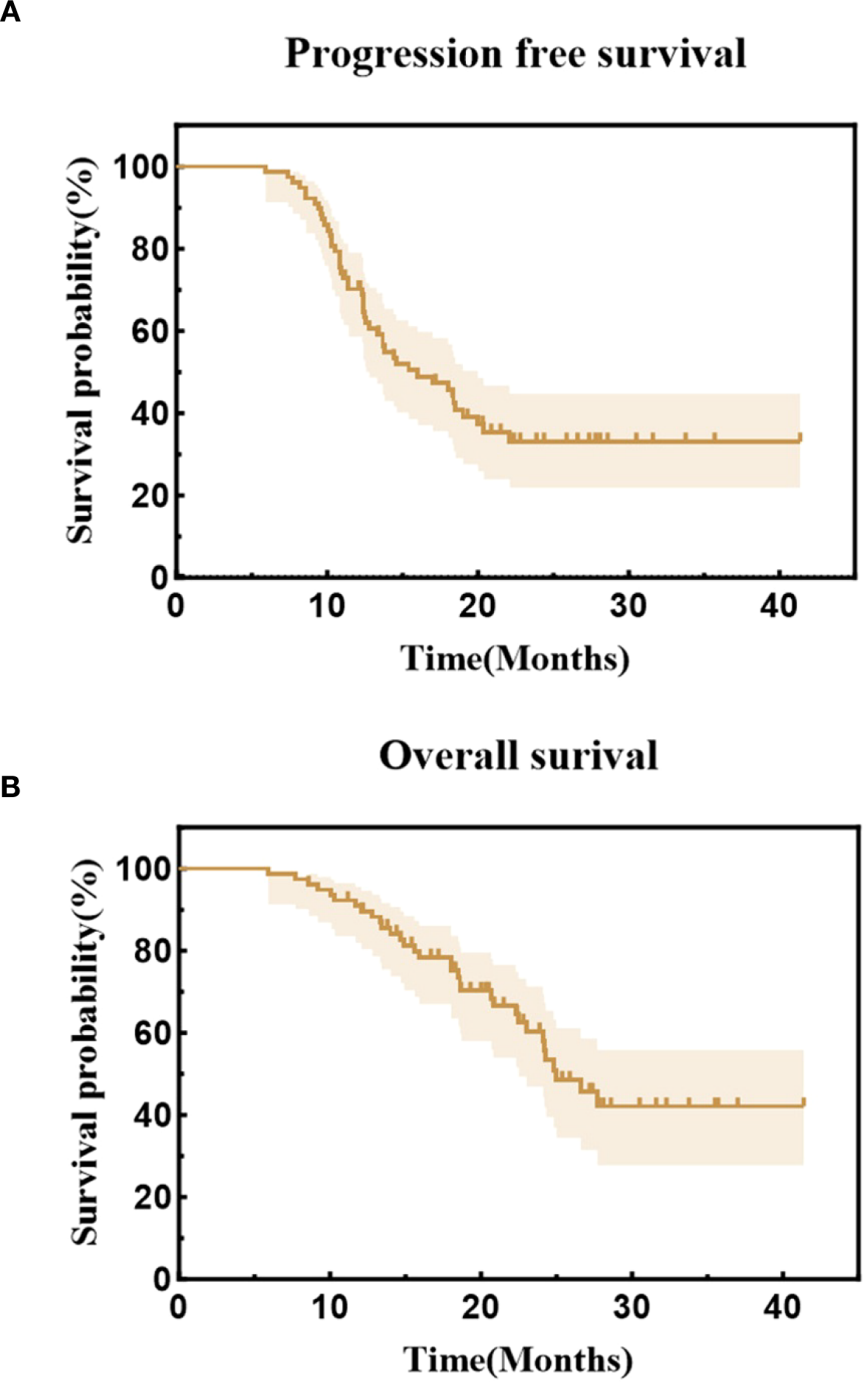
**(A)** Kaplan-Meier curves of progression-free survival for the entire cohort. **(B)** Kaplan-Meier curves of overall survival for the entire cohort.

**Figure 3:**
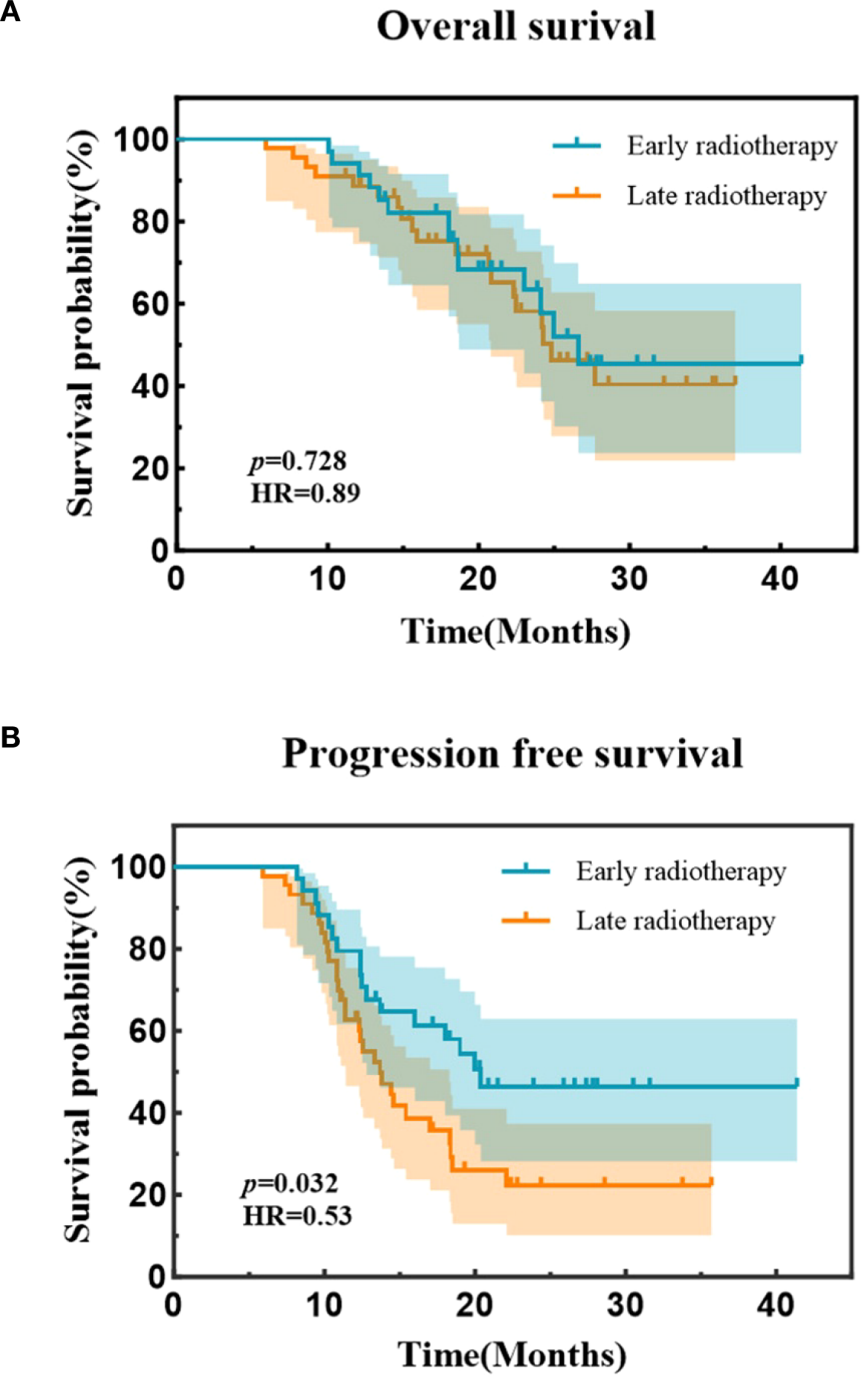
**(A)** Kaplan-Meier curves of progression-free survival between two groups. **(B)** Kaplan-Meier curves of overall survival between two groups.

**Figure 4:**
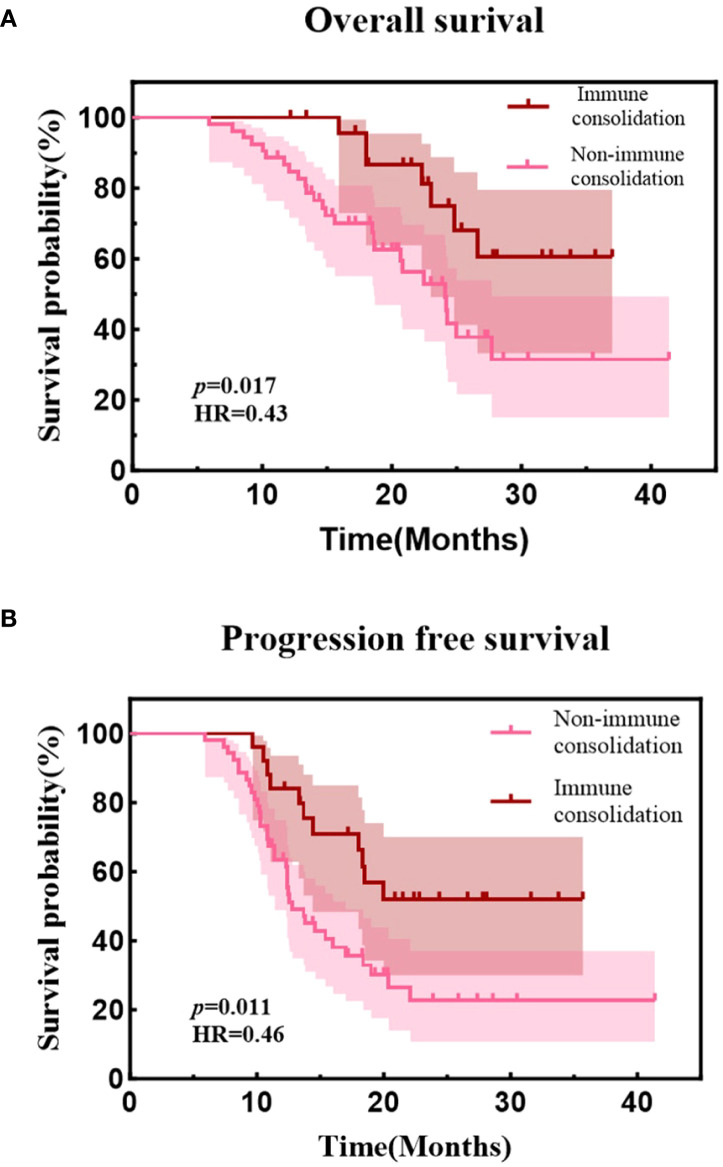
**(A)** Kaplan-Meier curves of progression-free survival between consolidation group and the non-consolidation group. **(B)** Kaplan-Meier curves of overall survival between consolidation group and the non-consolidation group.

As of the follow-up cutoff date (May 31, 2025), the median follow-up duration was 25.9 months (range, 5.9-41.4 months). The median PFS and OS for the entire cohort were 16.0 months (95% CI, 11.7-20.3) and 25.0 months (95% CI, 21.49-28.51), respectively. The estimated 1-year and 2-year PFS rates were 70.2% and 33.1%, while the estimated 1-year and 2-year OS rates were 90.9% and 60.3%. (**Figures 2A, B**). Median PFS was 20.4 months in the early radiotherapy group vs. 13.7 months in the late radiotherapy group, with 1-year PFS rates of 79.4% vs. 62.8%, and 2-year PFS rates of 46.5% vs. 22.3% (HR = 0.53; *p* = 0.032). Median OS was 26.6 months vs. 24.8 months, with 1-year OS rates of 94.1% vs. 88.5%, and 2-year OS rates of 63.5% vs. 58.1% (HR = 0.89; *p* = 0.728). (**Figures 3A, B**). In a subgroup analysis of the 25 out of 78 patients who received consolidation immunotherapy following radiotherapy. Median PFS was not reached (NR) in the consolidation group vs. 12.8 months in the non-consolidation group, with 1-year PFS rates of 84.0% vs. 63.5%, and 2-year PFS rates of 52.0% vs. 22.6% (HR = 0.46; *p* = 0.011). Median OS was NR vs. 24.1 months, with 2-year OS rates of 74.9% vs. 52.9% (HR = 0.43; *p* = 0.017). (**Figures 4A, B**).

### 3.3 Prognostic factors

**Figure 5:**
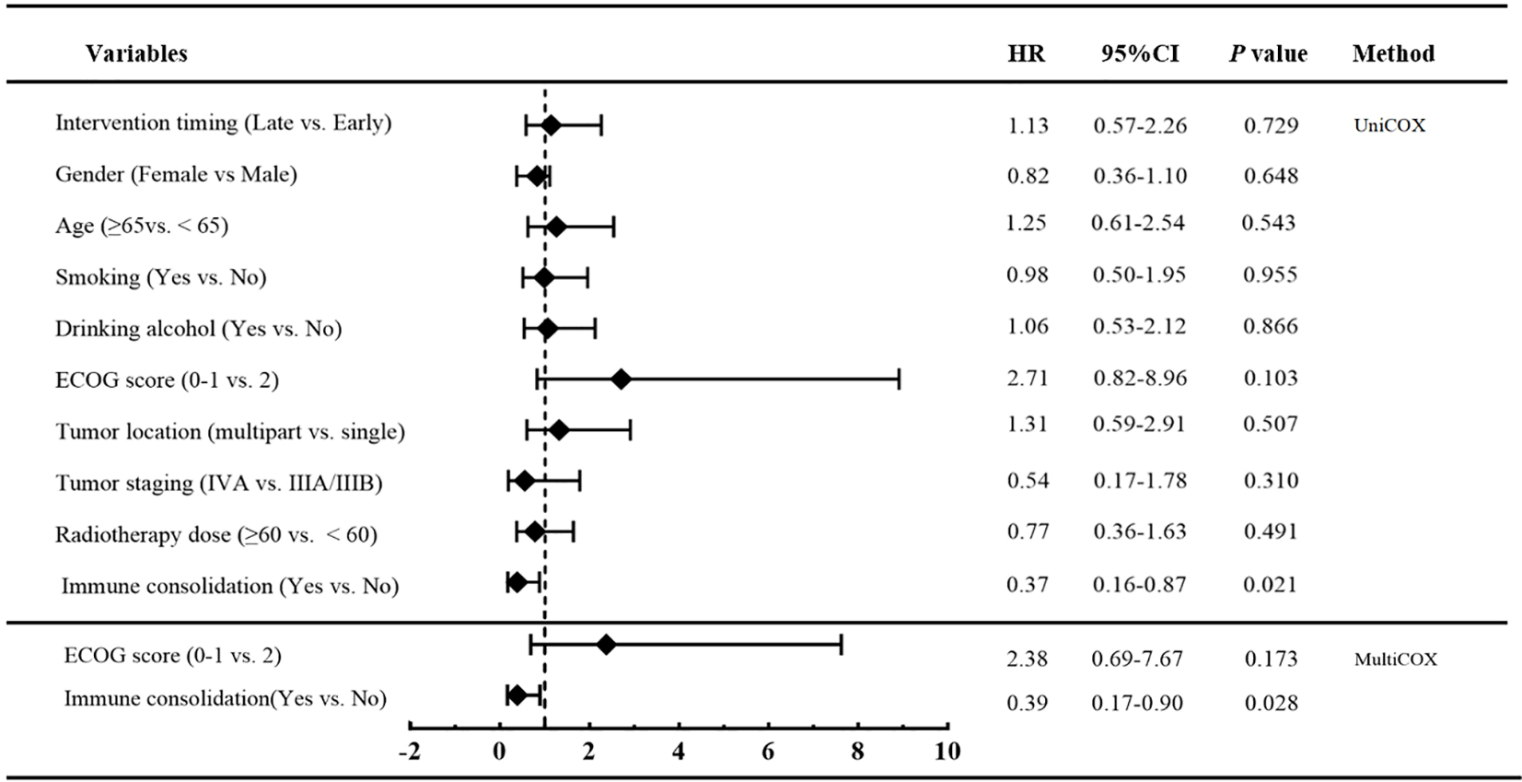
Univariate and multivariate cox regression analysis of PFS in patients in early radiotherapy group and late radiotherapy group.

Prognostic factors for PFS were assessed using univariate and multivariate Cox proportional hazards regression analyses, including timing of radiotherapy initiation, age, sex, smoking status, alcohol consumption, ECOG performance status, tumor location, clinical stage, radiotherapy dose, and receipt of immune consolidation therapy. Variables that showed statistical significance (p < 0.05) in the univariate analysis were included in the multivariate model. Multivariate analysis identified initiation of radiotherapy after more than 2 cycles of induction chemoimmunotherapy (HR = 1.91, 95% CI, 1.05-3.47, *p* = 0.035) and receipt of immune consolidation therapy (HR = 0.42, 95% CI, 0.22-0.84, *p* = 0.013) as independent prognostic factors for PFS. (**Figure 5**).

**Figure 6:**
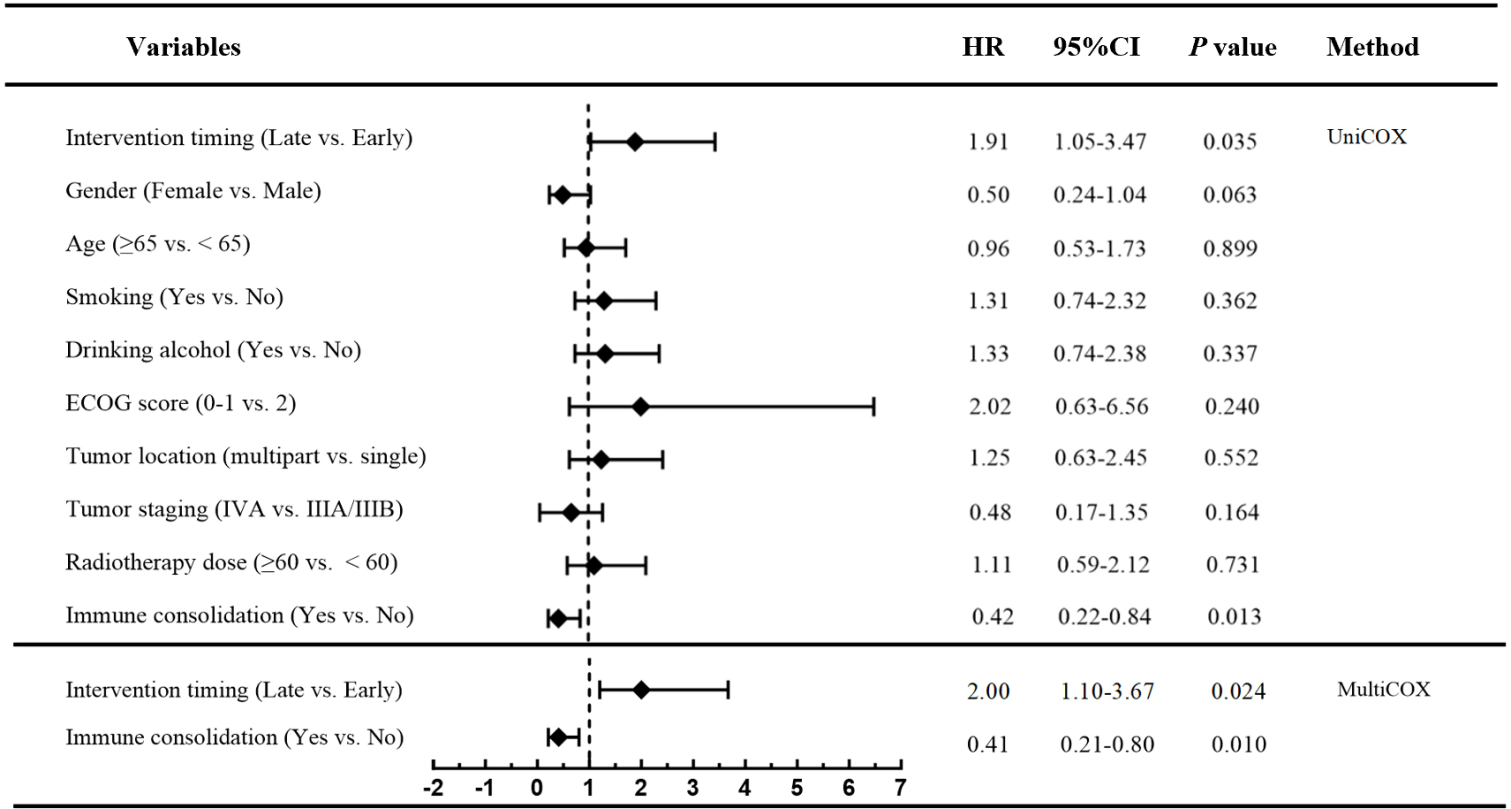
Univariate and multivariate cox regression analysis of PFS in patients in early radiotherapy group and late radiotherapy group.

Additionally, prognostic factors for OS were evaluated using univariate and multivariate Cox proportional hazards regression analyses. Variables with p-values < 0.20 in the univariate analysis were eligible for inclusion in the multivariate model. Multivariate analysis identified he receipt of immune consolidation therapy (HR = 0.39, 95% CI, 0.17-0.99, *p* = 0.028) as an independent favorable prognostic factor for OS. (**Figure 6**).

### 3.4 Treatment-related adverse event

Treatment-related adverse events (TRAEs) of any grade occurred in 78 patients. The most frequently reported TRAEs were radiation esophagitis (75 cases, 96.15%), anemia (68 cases, 87.18%), and leukopenia (53 cases, 67.95%), followed by thrombocytopenia (24 cases, 30.77%) and neutropenia (15 cases, 19.23%). Grade ≥3 TRAEs occurred in 25 patients (32.05%). These occurred in 7 patients in the early radiotherapy group and 18 patients in the late radiotherapy group (p = 0.098). One patient died from pneumonia, and two died from esophageal fistula. (**Table 2**).

**Table 2:** Treatment-related adverse events (TRAEs).

TRAEs	Any grade, n (%)			≥ 3级, n (%)		
	Early radiotherapy group	Late radiotherapy group	P value	Early radiotherapy group	Late radiotherapy group	P value
Leukopenia	21(58.82)	32(72.73)	0.304	2(5.88)	9(20.45)	0.132
Neutropenia	5(14.70)	10(22.73)	0.373	2(5.88)	3(6.82)	1.000
Thrombocytopenia	13(38.24)	11(25.00)	0.209	1(2.94)	4(9.10)	0.526
Anemia	29(85.29)	39(88.64)	0.923	1(2.94)	3(6.82)	0.801
Transaminase elevation	9(26.47)	6(13.64)	0.154	0(0.00)	0(0.00)	–
Thyroid dysfunction	2(5.88)	1(2.94)	0.840	0(0.00)	0(0.00)	–
Esophagitis	31(91.18)	44(100.00)	0.157	0(0.00)	5(11.36)	0.117
Pneumonitis	1(2.94)	2(4.55)	1.000	2(2.94)	0(0.00)	0.364

## 4 Discussion

To the best of our knowledge, this is the first study investigating the optimal timing of radiotherapy initiation following induction chemoimmunotherapy in the first-line treatment of patients with locally advanced unresectable ESCC. Our results demonstrate that, compared to the late radiotherapy group, the early radiotherapy group exhibited improved efficacy outcomes with a manageable safety profile.

Current clinical evidence suggests that patients with unresectable locally advanced ESCC achieve clinical benefit from a comprehensive therapeutic strategy involving induction chemoimmunotherapy followed by dCRT. This is evidenced by several studies: The ImpactCRT trial (6), utilizing two cycles of induction chemoimmunotherapy followed by dCRT, reported 1-year progression-free survival (PFS) and overall survival (OS) rates of 74.2% and 84.6%, respectively. The RICE study (7) demonstrated comparable outcomes, with 1-year PFS and OS rates of 76.0% and 92.9%, respectively. Similarly, data from a multicenter study (17) showed that patients receiving induction therapy achieved 1-year PFS and OS rates of 64.2% and 84.8%, respectively. Data from our institution revealed 1-year PFS and OS rates of 70.2% and 90.9%, respectively, which are consistent with the findings from the studies mentioned above (p > 0.05). This further corroborates the clinical value of induction chemoimmunotherapy followed by dCRT in managing unresectable locally advanced ESCC. Notably, the current body of evidence supporting this treatment approach primarily stems from phase I/II clinical trials or retrospective analyses, with a paucity of phase III randomized controlled trials (RCTs). Consequently, large-scale, multicenter, randomized phase III clinical trials are warranted to definitively establish the efficacy and safety profile of this therapeutic strategy. Furthermore, findings presented at the 2024 ASTRO Annual Meeting [18] demonstrated that consolidative immunotherapy following dCRT in locally advanced ESCC yielded a median PFS of 27.4 months and a median OS of 37.0 months, significantly surpassing outcomes historically achieved with dCRT alone (18). This study further investigated the benefit of consolidative immunotherapy. Our results indicate that for patients with unresectable locally advanced ESCC, the administration of consolidative immunotherapy after induction chemoimmunotherapy followed by radiotherapy was associated with significant PFS and OS benefits. These findings suggest that consolidative immunotherapy therapy following induction chemoimmunotherapy and radiotherapy has the potential to become an integral component of the standard treatment paradigm.

Currently, there is a paucity of definitive evidence regarding the optimal timing for radiotherapy initiation following induction chemoimmunotherapy and the identification of patient subgroups most likely to benefit in the management of unresectable locally advanced esophageal squamous cell carcinoma (ESCC). Preliminary findings from this study indicate that, compared to the late radiotherapy group, the early radiotherapy group achieved a significant improvement in PFS (20.4 months vs. 13.7 months, *p* = 0.032). This observed benefit is likely attributable to the efficacy of induction chemoimmunotherapy in reducing tumor volume and burden, with subsequent radiotherapy providing enhanced local control, thereby optimizing therapeutic outcomes (19). Previous murine models have also underscored the critical importance of therapeutic sequence, indicating that the administration of immunotherapy prior to radiotherapy is pivotal. The underlying mechanism for this temporal advantage is the preemptive abrogation of immunosuppression mediated by Tregs, thereby establishing a favorable immunogenic context for radiotherapy and potentiating its antitumor efficacy (20, 21). Furthermore, prolonging induction therapy to ≥3 cycles did not appear to confer additional improvement in tumor response rates. However, no significant difference in OS was observed between the groups (26.6 months vs. 24.8 months, *p* = 0.728). It is hypothesized that this may relate to the impact of post-progression therapies.

In this study, the most frequently observed TRAEs were radiation esophagitis and myelosuppression. Most of these events were grade 1 or 2 and were manageable with appropriate supportive care. The incidence of grade ≥3 radiation esophagitis was 6.8%, which is largely consistent with rates reported in prior studies (e.g., 5.9% in the RICE trial (7) and 5.63% in the study by Lian et al. (22)). Although there was no significant difference in the overall incidence of TRAEs between the early and late radiotherapy groups (*p* > 0.05), the incidence of myelosuppression was significantly higher in the late radiotherapy group than in the early radiotherapy group. This observation may be associated with the modulatory effects of chemotherapy and ICIs on the bone marrow microenvironment, impacting the survival, proliferation, and differentiation of hematopoietic stem cells (HSCs). Furthermore, a prior history of myelosuppression was identified as a significant risk factor for subsequent myelosuppression. These findings highlight the need for enhanced early monitoring and proactive management of myelosuppression in clinical practice.

This study has several limitations that should be acknowledged. First, as a single-center retrospective analysis with a limited sample size (n=78) and non-randomized group allocation, it is susceptible to selection bias and confounding factors, potentially compromising the robustness of the findings. Second, the relatively short median follow-up duration (25.9 months) may result in an overestimation of survival outcomes and an incomplete evaluation of late-onset TRAEs. Extending the follow-up period is therefore critical to definitively establish the long-term efficacy and safety profile of this treatment approach. Third, PD-L1 expression status was not routinely assessed in clinical practice during the study period. Consequently, we were unable to assess the correlation between PD-L1 expression levels and clinical outcomes (e.g., PFS or OS), which restricted the exploration of potential biomarkers for predicting response to immunotherapy. In conclusion, future multicenter, prospective randomized controlled trials (RCTs) are warranted to validate the present findings. Furthermore, such studies should incorporate comprehensive biomarker analyses (e.g., PD-L1, tumor mutational burden [TMB]) to elucidate their predictive value for treatment response.

## 5 Conclusion

In patients with unresectable locally advanced ESCC, a strategy combining early radiotherapy intervention with subsequent consolidative immunotherapy may represent a potential preferred therapeutic strategy. The findings from this study provide a rationale for the design of phase III clinical trials.

## Data Availability

The original contributions presented in the study are included in the article/supplementary material. Further inquiries can be directed to the corresponding author/s.

## References

[1] Bray F Laversanne M Sung H Ferlay J Siegel RL Soerjomataram I . Global cancer statistics 2022: GLOBOCAN estimates of incidence and mortality worldwide for 36 cancers in 185 countries. CA: A Cancer J Clin. (2024) 74:229–63. doi: 10.3322/caac.21834, PMID: 38572751

[2] Zhu H Ma X Ye T Wang H Wang Z Liu Q . Esophageal cancer in China: Practice and research in the new era. Int J Cancer. (2023) 152:1741–51. doi: 10.1002/ijc.34301, PMID: 36151861

[3] He J Chen WQ Li ZS Li N Ren JS Tian JH . China guideline for the screening, early detection and early treatment of esophageal cancer (2022, Beijing). Zhonghua Zhong Liu Za Zhi. (2022) 44:491–522. doi: 10.3760/cma.j.cn112152-20220517-00348, PMID: 35754225

[4] Cooper JS Guo MD Herskovic A Macdonald JS Martenson JA Al-Sarraf M . Chemoradiotherapy of locally advanced esophageal cancer: long-term follow-up of a prospective randomized trial (RTOG 85-01). Radiation Therapy Oncology Group. JAMA. (1999) 281:1623–1627. doi: 10.1001/jama.281.17.1623, PMID: 10235156

[5] Wang X Liang F Wang X Wu Y Wang D Cheng Y . Quality of life and survival outcomes of patients with inoperable esophageal squamous cell carcinoma after definitive radiation therapy: A multicenter retrospective observational study in China from 2015 to 2016. J Natl Cancer Cent. (2023) 3:150–8. doi: 10.1016/j.jncc.2023.05.001, PMID: 39035729 PMC11256718

[6] Peng F Bao Y Cheng C Niu S Song W Li Y . Induction chemotherapy plus camrelizumab followed by concurrent chemoradiotherapy in patients with unresectable locally advanced esophageal squamous cell carcinoma (ImpactCRT): A single-arm, phase II trial. J Clin Oncol. (2023) 41:e16067–e16067. doi: 10.1200/JCO.2023.41.16_suppl.e16067?af=R, PMID: 41271680 PMC12638862

[7] Lin L Li H Zhao L Cheng Y Liu MN Fu X . Induction immunochemotherapy followed by concurrent chemoradiotherapy for unresectable locally advanced esophageal squamous cell carcinoma (RICE): A prospective, single-arm, phase II trial. Int J Radiat OncologyBiologyPhysics. (2024) 120:e464–5. doi: 10.1016/j.ijrobp.2024.07.1032

[8] Ai D Hao S Shen W Wu Q Zhang S Chen Y . Induction sintilimab and chemotherapy followed by concurrent chemoradiotherapy for locally advanced esophageal cancer: a proof-of-concept, single-arm, multicenter, phase 2 trial. eClinicalMedicine. (2024) 69:102471. Available online at: https://www.thelancet.com/journals/eclinm/article/PIIS2589-5370(24)00050-6/fulltext (Accessed February 6, 2024)., PMID: 38356729 10.1016/j.eclinm.2024.102471 PMC10864194

[9] Liu F Wang D Luo Q Wu Y Guo J Zou Y . Neoadjuvant toripalimab plus chemotherapy followed by concurrent chemoradiotherapy in locally advanced esophageal squamous cell carcinoma (GASTO 1071). J Clin Oncol. (2024) 42:4077. doi: 10.1200/JCO.2024.42.16_suppl.4077

[10] Shah MA Bennouna J Doi T Shen L Kato K Adenis A . KEYNOTE-975 study design: a Phase III study of definitive chemoradiotherapy plus pembrolizumab in patients with esophageal carcinoma. Future Oncol. (2010) 17:1143–53. doi: 10.2217/fon-2020-0969, PMID: 33533655 PMC7927908

[11] Zhang W Zhang T Chen X Li J Zheng Z Dong J . 1414P Iparomlimab and tuvonralimab (QL1706) with definitive chemoradiotherapy for locally advanced esophageal squamous cell carcinoma: An open-label phase II study. Ann Oncol. (2024) 35:S883. doi: 10.1016/j.annonc.2024.08.1480

[12] Wang X Kang X Zhang R Xue L Xu J Zhao X . Chemoradiotherapy and subsequent immunochemotherapy as conversion therapy in unresectable locally advanced esophageal squamous cell carcinoma: A phase II NEXUS-1 trial. Clin Cancer Res. (2024) 30:5061–72. doi: 10.1158/1078-0432.CCR-24-1236, PMID: 39544026

[13] Muto M Nomura M Sakanaka K Shimizu J Ohashi S Watanabe A . Effect of nivolumab combined with definitive chemoradiotherapy on response rate for esophageal cancer: An immune active intrinsic subtype could be its biomarker—The NOBEL trial. JCO. (2024) 42:4068–8. doi: 10.1200/JCO.2024.42.16_suppl.4068

[14] Yu R Wang W Li T Li J Zhao K Wang W . RATIONALE 311: tislelizumab plus concurrent chemoradiotherapy for localized esophageal squamous cell carcinoma. Future Oncol. (2021) 17:4081–9. doi: 10.2217/fon-2021-0632, PMID: 34269067

[15] Wang R Ling Y Chen B Zhu Y Hu Y Liu M . Long-term survival and *post-hoc* analysis of toripalimab plus definitive chemoradiotherapy for oesophageal squamous cell carcinoma: insights from the EC-CRT-001 phase II trial. eClinicalMedicine. (2024) 75:102806. doi: 10.1016/j.eclinm.2024.102806, PMID: 39281099 PMC11402426

[16] Bando H Kotani D Tsushima T Hara H Kadowaki S Kato K . TENERGY: multicenter phase II study of Atezolizumab monotherapy following definitive Chemoradiotherapy with 5-FU plus Cisplatin in patients with unresectable locally advanced esophageal squamous cell carcinoma. BMC Cancer. (2020) 20:336. doi: 10.1186/s12885-020-06716-5, PMID: 32312286 PMC7168951

[17] Yang X Wang X Xiao Q Ge X Yu N Li J . Definitive chemoradiotherapy combined with anti-PD-1 immunotherapy for inoperable esophageal squamous cell carcinoma: a multicenter real-world study. Cancer Biol Ther. (2025) 26:2504726. doi: 10.1080/15384047.2025.2504726, PMID: 40367097 PMC12080274

[18] Yu N Yang X Li J Feng G Zheng Z Deng L . Pacific” Modality in locally advanced esophageal squamous cell carcinoma: A propensity score-matched analysis of a real-world multicenter study. Int J Radiat OncologyBiologyPhysics. (2024) 120:e504–5. doi: 10.1016/j.ijrobp.2024.07.1120

[19] Tang L Chen L Xu G Zhang N Huang C Li W . Reduced-volume radiotherapy versus conventional-volume radiotherapy after induction chemotherapy in nasopharyngeal carcinoma: An open-label, noninferiority, multicenter, randomized phase 3 trial. CA Cancer J Clin. (2025) 75:203–15. doi: 10.3322/caac.21881, PMID: 39970442 PMC12061627

[20] Young KH Baird JR Savage T Cottam B Friedman D Bambina S . Optimizing timing of immunotherapy improves control of tumors by hypofractionated radiation therapy. PLoS One. (2016) 11:e0157164. doi: 10.1371/journal.pone.0157164, PMID: 27281029 PMC4900555

[21] Bos PD Plitas G Rudra D Lee SY Rudensky AY . Transient regulatory T cell ablation deters oncogene-driven breast cancer and enhances radiotherapy. J Exp Med. (2013) 210:2435–66. doi: 10.1084/jem.20130762, PMID: 24127486 PMC3804934

[22] Lian Hm Wu Jl Liufu Wj Yu Tt Niu Sq Bao Y . Induction immunotherapy plus chemotherapy followed by definitive chemoradiation therapy in locally advanced esophageal squamous cell carcinoma: a propensity-score matched study. Cancer Immunol Immunother. (2024) 73:55. doi: 10.1007/s00262-024-03649-x, PMID: 38366287 PMC10873219

